# Age-dependent phenotypic modulation of smooth muscle cells in the normal ascending aorta

**DOI:** 10.3389/fcvm.2023.1114355

**Published:** 2023-02-21

**Authors:** Brittany Balint, Inés García Lascurain Bernstorff, Tanja Schwab, Hans-Joachim Schäfers

**Affiliations:** Department of Thoracic and Cardiovascular Surgery, Saarland University Medical Center, Homburg, Germany

**Keywords:** smooth muscle cell (SMC), ascending aorta, phenotypic modulation, alpha smooth muscle actin (α-SMA), cellular senescence

## Abstract

**Objectives:**

Ascending aortic aneurysms are associated with pre-existing conditions, including connective tissue disorders (i.e., Marfan syndrome) and bicuspid aortic valves. The underlying mechanisms remain uncertain. Even less is known regarding ascending aortic aneurysms in individuals with normal (i.e., tricuspid) aortic valves (TAV), and without known aneurysm-associated disorders. Regardless of etiology, the risk of aortic complications increases with biological age. Phenotypic modulation of smooth muscle cells (SMCs) is a feature of ascending aortic aneurysms, whereby contractile SMCs are replaced with synthetic SMCs that are capable of degrading the aortic wall. We asked whether age itself causes dysfunctional SMC phenotype modulation, independent of aortic dilatation or pre-existing aneurysm-associated diseases.

**Methods:**

Non-dilated ascending aortic samples were obtained intra-operatively from 40 patients undergoing aortic valve surgery (range: 20–82 years old, mean: 59.1 ± 15.2). Patients with known genetic diseases or aortic valve malformations were excluded. Tissue was divided, and a portion was formalin-fixed and immunolabeled for alpha-smooth muscle actin (ASMA), a contractile SMC protein, and markers of synthetic (vimentin) or senescent (p16/p21) SMCs. Another fragment was used for SMC isolation (*n* = 10). Cultured SMCs were fixed at cell passage 2 and stained for phenotype markers, or were cultured indefinitely to determine replicative capacity.

**Results:**

In whole tissue, ASMA decreased (R^2^ = 0.47, *P* < 0.0001), while vimentin increased (R^2^ = 0.33, *P* = 0.02) with age. In cultured SMCs, ASMA decreased (R^2^ = 0.35, *P* = 0.03) and vimentin increased (R^2^ = 0.25, *P* = 0.04) with age. p16 (R^2^ = 0.34, *P* = 0.02) and p21 (R^2^ = 0.29, *P* = 0.007) also increased with age in SMCs. Furthermore, the replicative capacity of SMCs from older patients was decreased compared to that of younger patients (*P* = 0.03).

**Conclusion:**

By investigating non-dilated aortic samples from individuals with normal TAVs, we found that age itself has a negative impact on SMCs in the ascending aortic wall, whereby SMCs switched from the contractile phenotype to maladaptive synthetic or senescent states with increased age. Therefore, based on our findings, modification of SMC phenotype should be studied as a therapeutic consideration against aneurysms in the future, regardless of etiology.

## Introduction

The etiology of ascending aortic aneurysms is not fully understood. Although genetic and environmental risk factors play a role in a subset of cases, ascending aortic aneurysms occur mostly in the elderly (1), signifying age-related degeneration of the aortic wall. Age is a risk factor for ascending aortic aneurysms in the general population (2), and also in individuals already predisposed to aortic complications, such as those with a bicuspid aortic valve (3). Since studies of the human aorta are usually confounded by pre-existing clinical dilatation, understanding the role of age itself on aortic remodeling is not well-defined.

Imaging studies using multiphase CT and 3D MRI have shown that the ascending aorta stiffens, dilates and elongates with age (4–7). Histological studies have shown evidence of age-related aortic medial layer remodeling, including elastin degradation (8), and changes in smooth muscle cell (SMC) density (6) and morphology (9). SMCs generate contractile force, which is required for the structural and functional integrity of the aortic wall (10). Thus, age-related SMC dysfunction that impacts contractile proteins, such as alpha-smooth muscle actin (ASMA), may contribute to aortic remodeling. The majority of age-related SMC studies have been performed with the use of rat SMCs, which show inconsistent findings compared to human SMCs (11–13). Therefore, detailed studies of human vascular SMCs in the context of aging could help identify therapeutic targets in suppressing age-related cardiothoracic diseases. Primary human SMC studies are rare, however, due to the limited availability of normal aortic samples, especially from younger patients.

Previous studies have shown that SMCs can switch from their contractile phenotype to a synthetic state under stressful culture conditions, which is characterized by increased migration, proliferation and protein synthesis (14). Importantly, synthetic SMCs have been observed in aneurysmal aortas (15). Although synthetic SMCs are critical during development and in response to injury, their presence in the adult aorta is associated with degenerative remodeling due to their propensity for secreting ECM degrading enzymes (16). A third SMC phenotype which is less commonly observed in human aortas is cellular senescence. Senescent SMCs have a seno-destructive phenotype capable of remodeling surrounding extracellular matrix and essentially weakening the aortic wall (17). Whether age itself drives phenotypic modulation of SMCs from their normal contractile state to destructive synthetic or senescent phenotypes in normal-sized human aortas remains uncertain.

In the current study, we aimed to elucidate the effect of biological aging itself on SMC phenotypes in the ascending aortic wall. We first histologically evaluated phenotype markers in the aortic medial layer of individuals ranging from 20–82 years old. To confirm that our findings were SMC-specific, we then isolated ascending aortic SMCs from a subset of patients and evaluated their age-related phenotypes in culture. In order to clarify the effect of aging alone, independent of potential confounding causes of aortic wall degeneration, we only included patients who had non-dilated aortas and normal tricuspid aortic valves (TAV).

## Materials and methods

The data that support the findings of this study are available upon reasonable request. This study complies with the Declaration of Helsinki, and was carried out with approval from the regional Ethics Committee (Ständige Ethikkommission der Ärztekammer des Saarlandes, Proposal # 47/14). Written informed consent was obtained from all enrolled patients.

### Procurement of ascending aortic tissue

Ascending aortic specimens were obtained intra-operatively from 40 consecutive cardiac patients undergoing aortic valve surgery. A 4–5 mm wide circumferential strip of aortic tissue was excised 5–10 mm above the sinotubular junction from the anterior circumference of the thoracic aorta, adjacent to the aortotomy. Aortic valve morphology was determined pre-operatively by either trans-esophageal or trans-thoracic echocardiography, and only normal (i.e., tricuspid) aortic valves were included. Aortic valve morphology was confirmed intra-operatively by the surgeon. Aortic dimensions were determined pre-operatively by computed tomography, and confirmed intra-operatively by trans-esophageal echocardiography. Aortic samples extracted in the operating room were divided and were either immediately fixed in 4% phosphate-buffered formalin for histological studies, or were placed in PBS and directly transferred to a sterile tissue culture hood for immediate extraction of vascular SMCs.

### Exclusion criteria

Patients with dilated aortas of ≥4.0 cm were excluded from this study (18). Individuals with known connective tissue disorders through genetic testing or clinical signs of connective tissue disorders (e.g., Loeys-Dietz syndrome, Marfan Syndrome, etc.) or with chronic viral diseases (e.g., HIV, Hepatitis B/C) were also excluded. Aortic samples were evaluated microscopically, and those with evidence of inflammatory disease (e.g., atherosclerosis, aortitis) were omitted.

### Aortic SMC isolation and culture

Smooth muscle cells were cultured from the medial layer of human aortic samples by enzymatic digestion. To prepare an isolated sample of the media, the endothelial layer of the aorta was removed by scraping the luminal surface gently with a scalpel. The tissue was washed in PBS, and then the adventitia and outer-most medial layers were peeled away with forceps. The remaining medial fragment was washed with PBS, and then used for the enzymatic digestion.

The enzymatic solution was a combination of 830 μl of M199 Media (ThermoFisher Scientific, 11150059; +0.5% FBS + 1% penicillin/streptomycin), 150 μl of Liberase™ (Roche, 05401020001) and 30 μl of DNAse. The prepared aortic media sample was cut into ∼1 × 1 cm fragments and added to a 1.5 ml Eppendorf tube containing the enzymatic solution. The sample was then placed in a SMC incubator (37^°^C, 5% CO_2_, 95% humidity) for 2 h. After the incubation, the digested supernatant was collected through a cell strainer and kept on ice until after the final incubation period. The undigested tissue was placed in a new Eppendorf tube with fresh enzymatic solution. The process was repeated for a second digestion, with an incubation period of 1.5 h. The supernatant from the second digestion was combined with that of the first, and the solution was then centrifuged at 750 RPM for 6 min at 4^°^C. The ensuing cell pellet was reconstituted in M199 media (+10% FBS + 1% penicillin/streptomycin), and the cells were plated on 0.4% gelatin-coated 60 mm cell culture dishes (Gelatin Type B Powder, Sigma-Aldrich, G9391; ThermoFisher Scientific Cell Culture Petri Dishes, 150340). The SMCs were maintained in the SMC incubator, as above.

The SMC media was changed 24 h after the isolation was completed, and then every 2 days until the cells reached confluence. When confluence was reached, the cells were trypsinized (Trypsin/EDTA solution, ThermoFisher Scientific, R001100) and plated on coverslips for immunocytochemistry (cell passage 1), or were plated on a fresh culture dish for continued growth and subsequent analysis of cellular senescence. For immunocytochemistry, serum was withdrawn from culture when the cells reached 80% confluence, and the cells were fixed with 4% paraformaldehyde after 72 h. For cellular senescence studies, the cells were re-platted each time confluence was reached until the cells stopped growing.

### Aortic tissue and cell immunostaining

Formalin-fixed, paraffin embedded whole aortic tissue samples were sectioned at 1 μm thickness. Aortic tissue sections and paraformaldehyde-fixed primary aortic SMCs were immunolabeled with polyclonal rabbit antibodies against ASMA (1:100, ab5694, Abcam), which was used as a marker of contractile stress fibers, or vimentin (1:100, ab137321, Abcam), a marker of synthetic SMCs (19). To assess for cellular senescence, aortic sections and SMCs were immunolabeled with monoclonal mouse antibodies against p16INK4a (1:50, MA5–17054, Invitrogen) and p21 (1:50, MA1–33926, Invitrogen), cyclin-dependent kinase inhibitors that delineate two core senescence initiation pathways (20, 21). Bound primary antibodies were visualized using fluorescent-conjugated secondary antibodies: goat anti-rabbit Alexa–594 (for ASMA and vimentin), or goat anti-mouse Alexa–594 (for p16 and p21). Aortic sections were counterstained with DAPI before mounting. For the SMCs, coverslips were mounted with DAPI-containing mounting media (Vectashield^®^, H–1200–10).

### Microscopy and image analysis

Fluorescent images were captured using a laser scanning confocal microscope (Zeiss LSM, Plan Apochromat). Tissue sections were imaged at 40× with a 1.3 oil objective. For the SMCs, images were captured at 20× (0.8 M27 objective). For each patient sample, five regions of interest (ROI) were captured per stain. For aortic tissue and cells, fluorescence intensity of ASMA and vimentin were measured with ImageJ (NIH), and were normalized to the background fluorescence intensity of the sample area. For p16 and p21, % positive was calculated by counting the number of positive nuclei and normalizing by the number of total nuclei per sample.

### Statistics

All statistical analyses were carried out using Prism 9 (Graphpad Software). The D’Agostino and Pearson omnibus test was applied to all data sets to test for normality. All data sets were normally distributed, except for replicative capacity for the “old age” patient group in Figure 3. Therefore, replicative capacity comparisons between age groups were carried out using the Mann-Whitney U test (for non-normal distributions). Data are presented as median ± interquartile range. For all other data sets, the student’s *t*-test was used for comparisons between groups. Mean ± standard deviation (SD) are presented. Linear regression analyses (for normal distributions) were used to assess relationships between continuous variables. Significance was set at *P* < 0.05.

**FIGURE 1:**
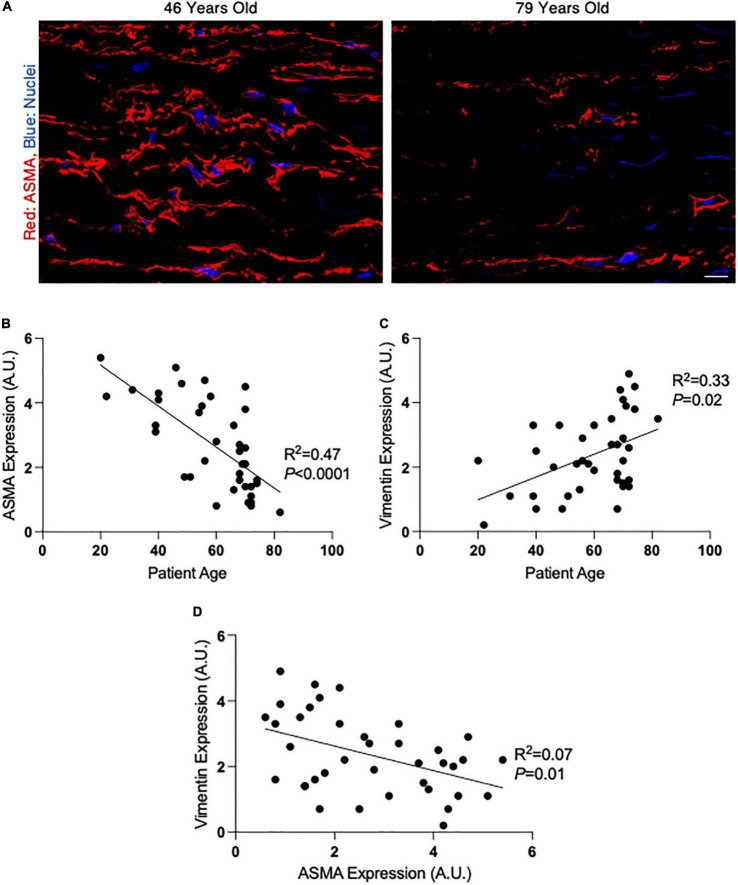
Alpha-smooth muscle actin (ASMA) decreases and vimentin increases with age in non-dilated ascending aortas. **(A)** Fluorescent micrographs of ascending aortic cross-sections from a 46 year old (left) and 79 year old (right) patient. Red, ASMA; blue, nuclei. Scalebar = 20 μm. **(B,C)** Graphs depicting the linear relationship between patient age and ASMA **(B)** or Vimentin **(C)** expression in non-dilated aortic tissue. **(D)** Graph depicting the relationship between ASMA and vimentin expression in non-dilated aortic tissue. A.U., arbitrary units; expression values were calculated by measuring fluorescence intensity.

**FIGURE 2:**
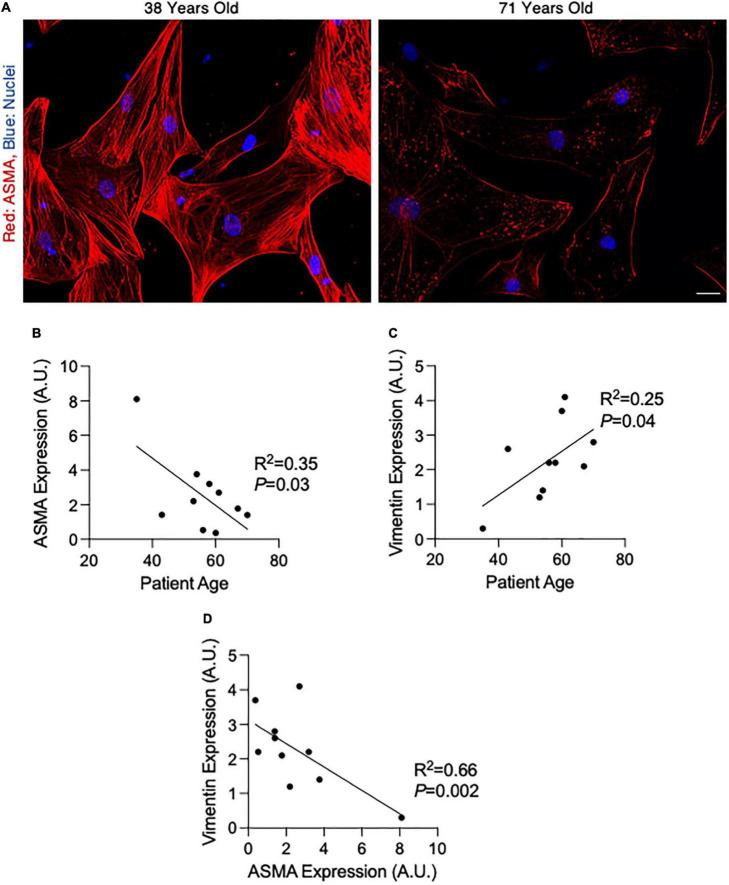
Alpha-smooth muscle actin (ASMA) decreases and vimentin increases with age in smooth muscle cells (SMCs) isolated from non-dilated ascending aortas. **(A)** Fluorescent micrographs of cultured SMCs from a 38 year old (left) and 71 year old (right) patient. Red, ASMA; blue, nuclei. Scalebar = 10 μm. **(B,C)** Graphs depicting the linear relationship between patient age and ASMA **(B)** or Vimentin **(C)** expression in cultured aortic SMCs. **(D)** Graph depicting the relationship between ASMA and vimentin expression in cultured aortic SMCs. A.U., arbitrary units; expression values were calculated by measuring fluorescence intensity.

**FIGURE 3:**
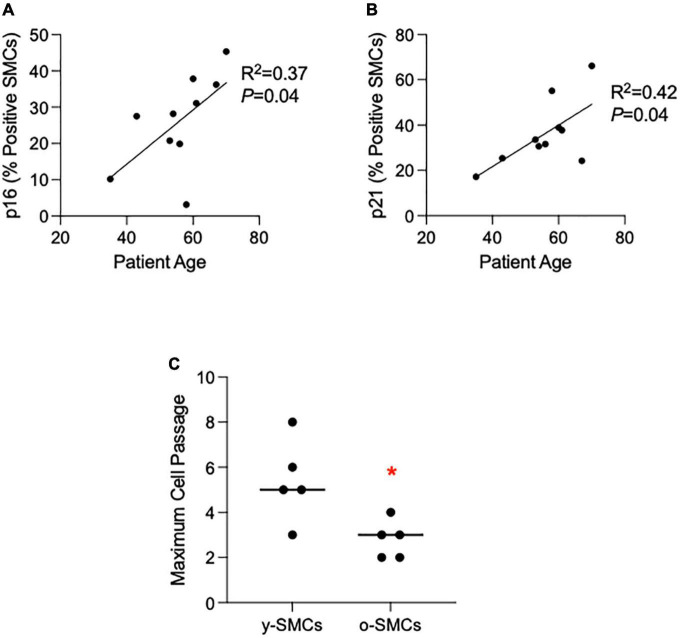
Cellular senescence increases with age in smooth muscle cells (SMCs) isolated from non-dilated ascending aortas. **(A,B)** Graphs depicting the linear relationship between patient age and the percentage of p16- **(A)** or p21- **(B)** positive SMCs in culture. **(C)** Graph depicting the maximum cell passage for SMCs from either young (y-SMCs; <55 years old) or older (o-SMCs; ≤55 years old) patient aortas. Comparison was made *via* Mann Whitney U Test. *P = 0.03. Horizontal line represents median values.

## Results

### Clinical characteristics

Ascending aortic tissue was extracted intra-operatively from a total of 40 patients (range: 20–82 years old, mean: 59.1 ± 15.2). All patients had TAVs and aortic dimensions within the clinically normal range (2.5–4.0 cm, mean: 3.3 ± 0.6 cm). Of the 40 patients, 17 presented with primary aortic regurgitation (AR), 18 presented with primary aortic stenosis (AS), and 5 showed a mixed pathology of both AR and AS. Patients with AS were significantly older than those with AR (*P* = 0.02; Supplementary Figure 1A). Clinical characteristics are summarized in Table 1.

**TABLE 1:** Patient characteristics.

	Whole aortic tissue	SMCs
Number of patients	40	10
Patient age (Range)	20–82	35–71
Patient age (Mean ± SD)	59.1 ± 15.2	55.7 ± 10.5
Ascending aortic diameter (Range; cm)	2.5–4.0	2.5–4
Ascending aortic diameter (Mean ± SD; cm)	3.3 ± 0.6	3.1 ± 0.4
**Aortic valve pathology**		
Regurgitation	17	4
Stenosis	18	4
Combined	5	2
**Co-morbidities; *n* (%)**		
Hypertension	18 (45)	4 (40)
Hyperlipidemia	4 (10)	1 (10)
**Medications; *n* (%)**		
ß-Blocker	15 (38)	3 (30)
ACE inhibitor	10 (25)	3 (30)
Diuretic	12 (30)	2 (20)
Calcium channel blocker	4 (10)	0
Statin	9 (23)	3 (30)

SMCs, smooth muscle cells; SD, standard deviation; ACE, angiotensin converting enzyme.

### SMC phenotypic modulation with age in aortic tissue

In whole aortic tissue samples, markers of contractile and synthetic SMCs were assessed by fluorescent immunohistochemistry. We found that the expression of ASMA, a contractile marker, decreased with age in the medial layer of ascending aortic tissue (fluorescence intensity: R^2^ = 0.47, *P* < 0.0001; Figures 1A, B). On the other hand, the expression of vimentin, a marker of synthetic SMCs, increased with age in aortic tissue samples (fluorescence intensity: R^2^ = 0.33, *P* = 0.02; Figure 1C). We found a significant inverse correlation between ASMA and vimentin in whole aortic samples (R^2^ = 0.07, *P* = 0.01; Figure 1D), suggesting an age-dependent modulation from the contractile to the synthetic phenotype in the normal ascending aorta. ASMA and vimentin expression were evaluated in aortic tissue from individuals with AS versus AR, and no differences were observed (*P* = 0.94 and *P* = 0.90, respectively; Supplementary Figure 1B).

### SMC senescence increases with age in the normal-sized ascending aorta

To assess for cellular senescence in the aortic media, we immunostained for the cell cycle inhibitors, p16 and p21. We found co-labeling of p16 and p21 in a small but significant portion of ascending aortas (15%). Interestingly, p16 and p21-positive SMCs were observed together only in aortas from patients 69 years of age or older. In patients younger than 69 years old, 2 patients had positive p16 staining and 5 patients had positive p21 staining. There was no evidence of double p16 and p21 positive staining in ascending aortas from individuals younger than 69 years old. These results suggest the possibility of age-associated increases in cell cycle inhibitor expression and possible SMC senescence in the normal ascending aorta.

### Cultured SMCs exhibit age-dependent phenotypic modulation

To determine whether medial SMCs themselves harbor age-related changes that could influence the integrity of the ascending aortic wall, we isolated SMCs from 10 aortic samples and studied them in culture. Similar to findings in whole tissue, ASMA expression significantly decreased with age (R^2^ = 0.35, *P* = 0.03; Figures 2A, B) and vimentin increased with age in cultured SMCs (R^2^ = 0.25, *P* = 0.04; Figure 2C). Accordingly, there was a significant negative correlation between ASMA and vimentin in cultured SMCs (R^2^ = 0.66, *P* = 0.002; Figure 2D) that is apparently influenced by patient age.

### Cultured SMCs enter senescence earlier with increased patient age

In order to assess for SMC senescence, we analyzed expression levels of p16 and p21, and we tested the replicative capacity of SMCs in culture. In contrast to whole aortic samples, the expression of both p16 and p21 were present in cultured SMCs from all patients. Interestingly, both p16 (R^2^ = 0.37, *P* = 0.04) and p21 (R^2^ = 0.42, *P* = 0.04) expression increased with patient age (Figures 3A, B). The mean patient age for SMC studies was 55.7 ± 10.5. Therefore, to test for age-related differences in SMC replicative capacity, we grouped SMCs into 2 categories: SMCs from patients < 55.7 years old [young (y)-SMCs; *n* = 5, patient age: 45 ± 5.4] and SMCs from patients ≥ 55.7 years old [old (o)-SMCs; *n* = 5, patient age: 68 ± 7.2]. By allowing cultured SMCs to grow indefinitely, we found that y-SMCs (cell passage: 5.0 ± 3.0) had an increased replicative capacity compared to o-SMCs (cell passage: 3.0 ± 1.5; *P* = 0.03; Figure 3C). These results suggest that ascending aortic SMCs enter senescence more rapidly with increased patient age.

## Discussion

Dilatation of the ascending aorta places the patient at risk for aortic complications (e.g., dissection, rupture, and sudden death). A relevant number of thoracic aortic aneurysms have known etiologies, including monogenetic syndromes (e.g., Marfan and Loeys-Dietz syndromes) and congenital aortic valve malformations (e.g., bicuspid or unicuspid aortic valves). Importantly, regardless of etiology, the prevalence of ascending aortic aneurysms increases with age (22). Therefore, a factor important to the maintenance of aortic wall integrity must experience an age-related decline or change. Studying the effect of aging alone on the human aorta, however, is difficult to achieve as surgical samples are normally confounded by dilatation and/or by pre-existing diseases that initiate the need for surgical repair.

### Phenotypic switching of SMCs in the non-dilated aorta

In order to investigate the relative effect of age without concomitant aneurysm, we evaluated aortic tissue samples from 40 individuals with non-dilated aortas and normal TAVs. We investigated SMC phenotypes in the normal aorta, as previous studies have shown that SMCs within the aneurysmal ascending aorta demonstrate a switch from the contractile to the synthetic state (23). The function and integrity of the aortic wall is maintained by the contractile force of vascular SMCs (10). In response to various pathological stimuli, however, SMCs switch to the synthetic phenotype where they are migratory, proliferative and prone to extracellular matrix remodeling (24, 25). Thus, phenotypic switching of SMCs leads to vascular dysfunction. In the current study, we found a similar phenotypic switch of SMCs. Interestingly, this was related to increased patient age, with a marked decrease in ASMA expression and increased vimentin expression. Therefore, our data suggest that SMC phenotypic switching may occur prior to marked aortic degeneration, and not solely as a pathological consequence of dilatation. It is also important to note that in the current study, the patients with predominant aortic stenosis were, on average, older than those with aortic regurgitation. Although no significant differences were observed in SMC phenotypes related to aortic stenosis or regurgitation, future age-matched studies should evaluate how different flow characteristics due to aortic valve dysfunction could impact SMC phenotypes in the ascending aortic wall.

### Phenotypic switching of cultured SMCs from the non-dilated aorta

To further investigate the age response in aortic SMCs, we isolated and cultured SMCs from the aortas of 10 patients. In younger patients, ASMA expression was high, and decreased with increasing patient age. Similar results were found in SMCs isolated from skeletal muscle feed arteries in young and old rats (26). As well, in comparison to healthy aortic SMCs, another study found that SMCs isolated from aneurysmal human aortas had decreased ASMA (27), indicating SMC phenotypic switching in remodeling aortas. We also found that vimentin increased with patient age in cultured SMCs, suggesting a higher prevalence of synthetic SMCs in aging aortas (19). Importantly, signals derived from endothelial cells and the extracellular matrix can have a profound impact on SMC differentiation (28). By isolating SMCs from the aorta, we confirmed that our results are SMC-specific, and independent from on-going influences from the surrounding aortic tissue. Our findings suggest that age itself is related to SMC phenotypic modulation, a process that may underlie aortic dilatation and/or exacerbate dilatation that is derived from other etiologies.

### SMC senescence

A less common SMC phenotype that has recently been identified in aneurysmal aortas is cellular senescence. SMC senescence is characterized by a state of essentially permanent cell cycle arrest with ongoing metabolic activity, including a shift in the secretome. Previous studies of mice (29) and humans (17) have identified a population of senescent SMCs in the aneurysmal ascending aorta. In the current study, we found that p16 and p21 expression increased with age in the aorta and in cultured SMCs, which may be indicative of senescent SMCs. This was further supported by our finding of decreased replicative capacity of cultured SMCs from older versus younger patients. Importantly, recent evidence suggests that the senescence-associated secretory phenotype has detrimental effects on the aortic wall, particularly due to the increased expression of matrix degrading enzymes (17). These findings suggest that SMCs in the aortic media undergo age-related cellular senescence, even in the absence of aortic dilatation. Whether senescence occurs in the aging aorta as a less common fate of phenotypic modulation, or whether it occurs as an independent age-related stress-response remains to be explored.

In summary, our findings suggest that age itself has a negative impact on the ascending aortic wall, particularly through phenotypic modulation of vascular SMCs. Although previous studies have shown similar findings in aneurysmal aortas, the current study is the first to investigate the effect of age on non-dilated aortas from individuals without known aneurysmal disorders. These findings suggest the need for careful consideration of therapeutic targets for ascending aortic aneurysms. Modification of SMC phenotype may theoretically be studied as a therapeutic consideration in the future.

## Data availability statement

The raw data supporting the conclusions of this article will be made available by the authors, without undue reservation.

## Ethics statement

The studies involving human participants were reviewed and approved by Ethikkommission bei der Ärztekammer des Saarlandes, No. 205/10, Date of Issue: 08.12.2010. The patients/participants provided their written informed consent to participate in this study. Written informed consent was obtained from the individual(s) for the publication of any potentially identifiable images or data included in this article.

## Author contributions

BB conceptualized the project, collected the data, supervised, wrote, and edited the manuscript. IB collected and analyzed data. TS performed the experiments and provided the resources. H-JS supervised, provided the resources, and edited the manuscript. All authors contributed to the article and approved the submitted version.
