# Supplementary figures and images for: Age-dependent phenotypic modulation of smooth muscle cells in the normal ascending aorta
Source: Front Cardiovasc Med. 2023 Feb 21;10:1114355. doi: 10.3389/fcvm.2023.1114355 (PMC9989028; doi:10.3389/fcvm.2023.1114355)

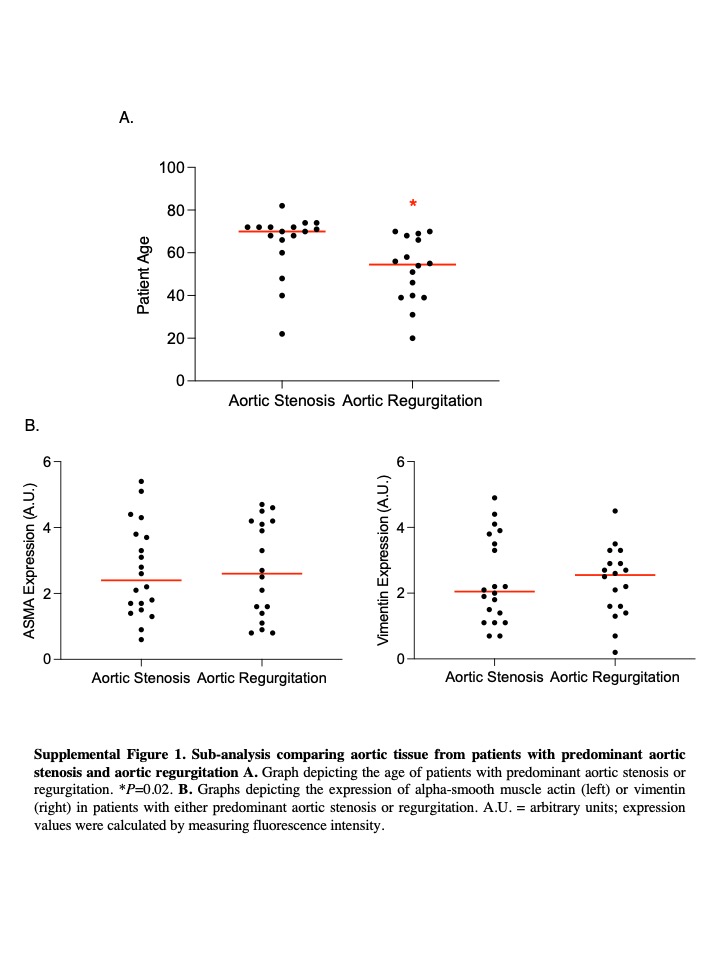

Supplement: Supplementary file 1 [file Image_1.jpg]
